# Breath Analysis Using Laser Spectroscopic Techniques: Breath Biomarkers, Spectral Fingerprints, and Detection Limits

**DOI:** 10.3390/s91008230

**Published:** 2009-10-19

**Authors:** Chuji Wang, Peeyush Sahay

**Affiliations:** Department of Physics and Astronomy and The Institute for Clean Energy Technology, Mississippi State University, Starkville, MS 39759, USA

**Keywords:** breath analysis, biomarkers, laser spectroscopic detection techniques, TDLAS, CRDS, ICOS, CEAS, CALOS, PAS, OFC-ECAS, acetone, nitric oxide, carbon dioxide

## Abstract

Breath analysis, a promising new field of medicine and medical instrumentation, potentially offers noninvasive, real-time, and point-of-care (POC) disease diagnostics and metabolic status monitoring. Numerous breath biomarkers have been detected and quantified so far by using the GC-MS technique. Recent advances in laser spectroscopic techniques and laser sources have driven breath analysis to new heights, moving from laboratory research to commercial reality. Laser spectroscopic detection techniques not only have high-sensitivity and high-selectivity, as equivalently offered by the MS-based techniques, but also have the advantageous features of near real-time response, low instrument costs, and POC function. Of the approximately 35 established breath biomarkers, such as acetone, ammonia, carbon dioxide, ethane, methane, and nitric oxide, 14 species in exhaled human breath have been analyzed by high-sensitivity laser spectroscopic techniques, namely, tunable diode laser absorption spectroscopy (TDLAS), cavity ringdown spectroscopy (CRDS), integrated cavity output spectroscopy (ICOS), cavity enhanced absorption spectroscopy (CEAS), cavity leak-out spectroscopy (CALOS), photoacoustic spectroscopy (PAS), quartz-enhanced photoacoustic spectroscopy (QEPAS), and optical frequency comb cavity-enhanced absorption spectroscopy (OFC-CEAS). Spectral fingerprints of the measured biomarkers span from the UV to the mid-IR spectral regions and the detection limits achieved by the laser techniques range from parts per million to parts per billion levels. Sensors using the laser spectroscopic techniques for a few breath biomarkers, e.g., carbon dioxide, nitric oxide, etc. are commercially available. This review presents an update on the latest developments in laser-based breath analysis.

## 1. Introduction

It is generally accepted that modern breath analysis started with the discovery made by Pauling in 1971 that hundreds of volatile organic compounds (VOCs) are present in normal human breath at the levels of parts per billion (ppb) or lower [1]. This discovery, together with other early breath studies [2], has regenerated research interest in human breath analysis for noninvasive disease diagnosis and metabolic status monitoring. Breath analysis can be classified into two groups: analysis of breath metabolites after administration of a drug or substrate and analysis of breath compounds produced endogenously due to a particular physiological status. Normal human breath contains a few atmospheric molecules, e.g., H_2_O, CO_2_, N_2_, O_2_, in relatively high concentrations, several VOCs, e.g., acetone, isoprene, propanols, etc., at the parts per million (ppm) or sub ppm levels, and about four hundred major VOCs (of more than 1,000 breath compounds) at the ppb or parts per trillion (ppt) levels [1,3,4]. To date, some VOCs have been established as biomarkers for specific diseases or metabolic disorders. For instance, alkanes are present in the case of lung cancer and formaldehyde in the case of breast cancer; the presence of isoprene in human breath is related to blood cholesterol levels; and patients with Type 1 diabetes have excess acetone in their breath. Such knowledge suggests that breath analysis is useful for human disease diagnosis and/or metabolic status monitoring. However, due to the low concentrations and large quantity of trace compounds in exhaled breath, breath analysis requires a highly sensitive and highly selective instrument in order to identify and determine concentrations of specific biomarkers. One major technique primarily employed for breath gas analysis is gas chromatography-mass spectrometry (GC-MS). This method has a routine detection sensitivity of ppb to ppt and can analyze multiple compounds simultaneously and selectively; yet, GC-MS requires complicated procedures for sample collection and pre-concentration and also has high instrument costs [5-7]. Extensive studies have been conducted to identify and quantify breath biomarkers using GC-MS, to improve the methods of breath sample preparation, and to miniaturize devices [8-13]. However, current MS-based breath analyses are still limited to laboratory research, beyond the consideration of an affordable, real-time, point-of-care (POC) clinical instrument. In addition to conventional GC-MS methods, a relatively new technique, proton transfer reaction mass spectrometry (PTR-MS), has been used for breath profiling [14,15]. Vacuum-free ion mobility spectroscopy (IMS) combined with a multi-capillary column has also been used for identification of metabolites and bacteria in human breath [16,17]. IMS is slightly less sensitive than GC-MS and PTR-MS and shows much potential for the development of a hand-held breath device. By comparison, selected ion flow tube mass spectrometry (SIFT-MS), which also belongs to the MS-based category, performs exceptionally well in clinical breath analysis; on-line breath analysis of many breath compounds under various physiological conditions have been conducted in clinics with actual human breath [18-21]. Breath analysis is also conducted by using electrical sensors, which are comparatively inexpensive and smaller in size, but they have low detection selectivity and require frequent calibrations [22,23].

Recent advances in high-sensitivity, high-selectivity laser spectroscopic techniques as well as laser sources make it possible for breath analysis to advance from the MS-based, time-consuming, laboratory studies to laser-based, real-time, clinical testing [24,25]. Many breath biomarkers have been detected by the laser-based techniques, and detection sensitivities are comparable with those from MS-based measurements, e.g., ranging from the ppm to ppt levels. Several excellent reviews have discussed the current status, trends, and challenges of clinical breath analysis, the MS-based analytical techniques in breath analysis, and advanced laser techniques and laser sources in breath analysis [25-30]. This article gives an exhaustive review on the breath analysis of almost all of the biomarkers that have been analyzed to date in actual human breath using the high-sensitivity laser spectroscopic techniques. In addition, this article differentiates itself from previous reviews [25-30] by presenting a complete survey of spectral fingerprints, measuring techniques, and currently achieved detection limits of each of the biomarkers. Finally, in this review, the high-sensitivity laser spectroscopic techniques are restricted to the tunable diode laser absorption spectroscopy (TDLAS) [31,32], cavity ringdown spectroscopy (CRDS) [33-36], integrated cavity output spectroscopy (ICOS) [37-40], cavity enhanced absorption spectroscopy (CEAS) [41], cavity leak-out absorption spectroscopy (CALOS) [42-46], photoacoustic spectroscopy (PAS) [47], quartz-enhanced photoacoustic spectroscopy (QEPAS) [48], and optical frequency comb cavity-enhanced absorption spectroscopy (OFC-CEAS) [49,50]. The paper is structured as follow: Section 2 of this article lists the 35 major breath biomarkers which have been identified and quantified to date by all detection means, including MS-, chemical-, and laser-based techniques; in Section 3, the key principles of the TDLAS, CRDS, ICOS, CEAS, CALOS, PAS, QEPAS, and OFC-CEAS are briefly described with a common feature of their technological detection limits; Section 4 reviews the 14 major breath biomarkers that have been detected in actual human breath by the laser techniques to date; the current status of breath sensing instrumentation using the laser spectroscopic techniques is updated in Section 5; and challenges in the field of the laser spectroscopy based breath analysis are discussed in Section 6.

## 2. 35 Identified Breath Biomarkers and Their Related Physiological Symptoms

To date, more than 1,000 compounds have been identified to be present in exhaled human breath. Their concentrations range from ppb to ppt levels. Approximately, 35 of the identified compounds in the exhaled breath have been established as biomarkers for particular diseases and metabolic disorders. These biomarkers and their related diseases and metabolic disorders are listed in Table 1. Formation of these compounds is attributed to the biochemical reactions happening inside the body as a part of metabolic processes. For instance, acetone, which is produced normally in the body, primarily results from the spontaneous decarboxylation of the acetoacetate and to a lesser extent from enzymatic conversion of acetoacetate to acetone. Detailed formation mechanisms of some of the biomarkers and how each of them is related to a specific disease are not well understood. The relation between a biomarker and a specific disease is often multi-fold. In some cases, a breath species is a biomarker that is indicative of about more than one disease or metabolic-disorder; in other cases, one particular disease or metabolic disorder can be characterized by more than one chemical species. For example, breath nitric oxide (NO) is a biomarker for asthma, bronchieactasis, and rhinitis [51-54]; Ethane is a biomarker for vitamin E deficiency in children and an indicator of lipid peroxidation [55-57], and it serves as a bio-marker for asthma as well [58]. At the same time, hydrogen peroxide (H_2_O_2_) and ammonia (NH_3_) are also biomarkers for asthma [59,60]. Similarly, lipid peroxidation can be diagnosed through analysis of breath ethylene and pentane [61,62]. However, pentane together with carbon disulfide is a biomarker of schizophrenia [63]. These features require breath analysis to be not only highly-sensitive but also highly-selective in order to obtain accurate information.

Different techniques that are being used in the detection and measurement of breath compounds can be categorized into three major groups: GC-MS or other MS-based methods, laser absorption spectroscopic techniques, and electrical sensors. Detection limits of breath compounds using these techniques are in the range of ppm – ppt. Although all of these techniques have their own advantages and disadvantages, which have been intensively discussed in the literature [25-30], almost all of the breath compounds are initially identified and quantified by using the MS-based methods. Driven by the ultimate goals of noninvasive breath analysis for clinical application and home-owned POC medical instrumentation, laser spectroscopic breath analysis has begun to draw increasing attention during the last several years since advanced spectroscopic techniques, together with new laser sources, have made it possible to develop a POC instrument for online monitoring and analysis of human breath with high accuracy, sufficiently low detection limits, and affordable cost. The major laser spectroscopic techniques, which are characterized by their high sensitivities and are currently being used or underdevelopment for breath analysis, include TDLAS, CRDS, ICOS, CEAS, CALOS, PAS, QEPAS, and OFC-CEAS. The basic principles of these techniques are briefly described below.

## 3. Laser Spectroscopic Techniques for Breath Analysis

Tunable diode laser absorption spectroscopy (TDLAS) is an optical technique combining the conventional absorption spectral method with advanced tunable diode lasers [31,32]. In TDLAS, a sample gas is contained in a gas cell and a detector records the transmitted laser intensity as the laser wavelength is repetitively scanned through the central frequency ν of an absorption line of the gas. The relation between the incident laser intensity *I_0_* and the transmitted intensity *I* is expressed by the Beer-Lambert law:

(1) $$I(\nu )=I_{0}(\nu )e^{-\alpha (\nu )L}$$

where *L* is the optical path-length and *α(ν)* is the absorption coefficient at frequency ν. *α(ν)* is related to the absorption cross-section *σ(ν)* by:

(2) α(ν)=σ(ν)n

where *n* is the concentration of the sample gas (molecule cm^−3^). In many cases, the absorption line has a pressure- and temperature-dependent lineshape that is centered on the central frequency. Absorption is more accurately quantified by an integrated absorption over the entire absorption lineshape and expressed by a total single-pass absorbance *A*, which is defined as:

(3) $$A=\text{absorbance}=L\int_{0}^{\infty }\alpha (\nu )d\nu =nL\int_{0}^{\infty }\sigma (\nu )d\nu =\text{SnL}$$

where *S* is the line strength defined as:

(4) $$S=\int_{0}^{\infty }\sigma (\nu )d\nu$$

For a given transition line, the line strength is a function of gas temperature. Line intensities of spectral transitions of many important application-driven molecules are documented in the literature and have an accuracy of 1%–5% or better [64]. Equation (3) indicates that for a given experimental system (a fixed *L*), a transition line with a larger line strength *S* allows a lower detectable gas concentration *n*. Rearranging Equation (1) gives that the detection limit (DL) in terms of the minimum absorbance *A_min_* is determined by the minimum detectable fractional change of the laser intensity, Δ*I/I*_0_, where Δ*I* = *I*_0_ − *I*. The stability of the light source and electronic noise of the detector typically offer an *A_min_* on the orders of 10^−2^–10^−4^. Compared with this direct measurement of the intensity difference Δ*I*, TDLAS measurements are frequently carried out by using the harmonic detection technique, called wavelength modulation/frequency modulation spectroscopy (TDLAS-WM/FM) [65,66], in which a better signal-to-noise ratio resulting from the wavelength/frequency modulation scheme yields a lower detection limit (DL) for TDLAS-WM/FM of 10^−4^–10^−6^. If a multipass gas cell, e.g., a Herriott cell, is implemented in the TDLAS-WM/FM technique, DLs can be further lower. A Herriott cell is a multi-pass optical cavity which consists of two concave spherical mirrors separated by a fixed distance. The inlet beam is injected into the cell at a small angle through a hole in one of the mirrors. Under the condition of an appropriate alignment, the beam exits at an angle different from the injection angle through the same hole after multiple passes through the cell. In a typical case, the beam bounces back and forth between the two mirrors tracing an elliptical spot pattern on each mirror. Using a Herriott cell, the effective path-length can be typically increased up to tens to 100-fold. Thus DLs of the TDLAS-WM/FM technique combined with a Herriott cell can be as low as 10^−8^.

**Figure 1.**
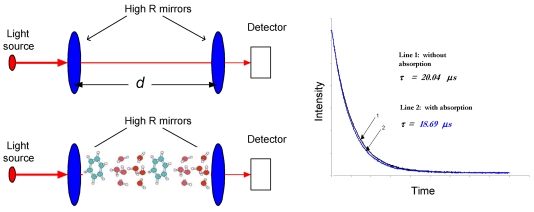
Illustration of the CRDS concept. The effective absorption path-length is readily increased more than 10,000-fold in CRDS. With and without absorption the decay time constants (ringdown times) are different; a ringdown decay example is shown in the right.

Cavity ringdown spectroscopy (CRDS) is a laser absorption technique [33-36], as pictorially in Figure 1. It obtains high sensitivities because of the multipass nature of the optical absorption path. It can be coupled to high resolution laser sources without adverse impacts from the intensity variation of the laser source. In CRDS, a laser beam is injected through one end-mirror of an optical cavity, where the injected light remains trapped between the mirror surfaces. The intensity of the light in the cavity decays exponentially with time at a rate determined by the round trip losses experienced by the laser pulse. These losses are typically due to the finite reflectivity of the cavity mirrors, optical absorption, and/or scattering. The decay behavior can be monitored by a photodetector located behind the second mirror.

In the simplest case, where there are no absorbing species present, the decay time (ringdown time), *τ*_0_, is determined by the speed of light, *c*, the mirror reflectivity, *R*, and the distance between the mirrors, *d*:

(5) $$\tau_{0}=\frac{d}{c(1-R)}$$

If a sample gas fills the optical cavity, there are additional losses due to absorption by the sample and the decay time is given by equation (6a). If the gas fills only a *portion* of the cavity, *l*, then the mirror separation, *d*, must be distinguished from *l* (the length of the absorption path), as in equation (6b):

(6a) $$\tau =\frac{d}{c(1-R+\sigma (\nu )nd)}$$

(6b) $$\tau =\frac{d}{c(1-R+\sigma (\nu )nl)}$$

Experimentally, one measures the ringdown lifetimes with absorption (*τ*) and without absorption (*τ*_0_). Thus, the absorbance can be rewritten as:

(7) $$A=\text{absorbance}=\sigma \left(\nu \right)nl=\frac{d}{c}(\frac{1}{\tau }-\frac{1}{\tau_{0}})$$

Equation (7) can be rearranged as:

(8) $$\sigma \left(\nu \right)nl=(1-R)\Delta \tau /\tau \;(\Delta \tau =\tau_{0}-\tau )$$

Given mirror reflectivities in the near infrared (NIR) of ∼99.9985 % and Δ*τ*/*τ* measurable to ∼10^−3^, the detection limit of the single-pass absorbance is on the order of 10^−8^.

Integrated cavity output spectroscopy (ICOS), cavity enhanced absorption spectroscopy (CEAS), and cavity leak-out absorption spectroscopy (CALOS) are the same technique but just named differently in the literature [37-40,41-46]. They are variants of the original CRDS technique and share the same multipass scheme to enhance sensitivity. The difference between these variants and CRDS is that, e.g., ICOS measures the integrated output of the light intensities with and without absorption of the species in the gas cell and the absorbance is determined by:

(9) $$\text{absorbance}=(1-R)\frac{\Delta I}{I_{0}}$$

**Figure 2.**
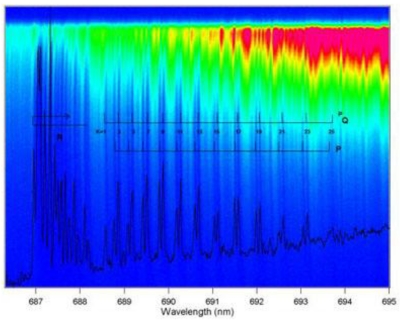
The combined time-domain and broadband ICOS spectra of the weak oxygen b-x (1,0) band is clearly seen. The broadband-ICOS spectrum can be recovered by doing a linear integration along a line at any constant height (the time axis in the Spectral Photography image) (reproduced with permission from the Optical Society of America [37]).

Upon comparison of Equation (8) with Equation (9), one finds that ICOS (or CEAS or CALOS) has similar detection sensitivity to CRDS. Although ICOS seems to be more robust in terms of the optical alignment requirements than CRDS, the determination of the mirror reflectivity in ICOS still requires CRDS measurements. With a broadband laser source, ICOS (or CEAS or CALOS) can measure several species concurrently or obtain a spectrum over a broad spectral window. Figure 2 shows the ICOS spectra of O_2_ obtained over a wide wavelength range.

**Figure 3.**
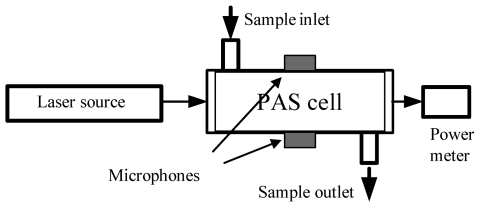
Schematic of photoacoustic spectroscopy.

Photoacoustic spectroscopy (PAS) differentiates itself from CRDS and the CRDS variants by using a sensitive microphone to sense acoustic waves launched by the absorption of pulsed or modulated radiation via transient localized heating and expansion in the absorbing medium. In PAS, as illustrated in Figure 3, the absorbing medium, e.g., a gas sample, in a specially designed PAS cell absorbs laser radiation at a selected frequency and the photon energy absorbed by the gas is translated into kinetic energy of the gas molecules or gas pressure variations through non-radiative relaxation processes. Unlike TDLAS and CRDS, PAS does not directly measure light absorption, but the detection selectivity of PAS for a gas species is still based on the resonance of the laser frequency with the selected transition frequency of the gas molecules. Based on the same photoacoustic effect, the acoustic waves generated from the absorption of the gas sample translates the acoustic energy to a narrow band acoustic transducer, e.g., a quartz tuning fork, and a resonant signal is detected when the acoustic wave is resonant with the vibration frequency of the fork; this new approach is named the quartz-enhanced PAS (QEPAS) [48]. In the original form of PAS for the detection of gas molecules, the detection limit (in terms of absorbance) is determined by the minimum detectable PAS signal (sound pressure), *P_min_*, laser power, *W_L_*, the setup constant of the PAS cell, *C*, and laser absorption path-length, *L*, which can be expressed as [67]:

(10) $$A_{\min}=\text{absorbance}=\alpha_{\min}(v)L=\frac{P_{\min}L}{CW_{L}}$$

For example, the background noise of a typical PAS detector is 100 nV–1 mV and the sensitivity of a typical PAS microphone is ∼100 mV/Pa. The minimum detectable sound pressure is in the range of 10^−5^–10^−7^ Pa. Given a setup constant of 2,000 Pa cm/W and absorption path-length of 10 cm, the detection limit, *A_min_*, of the PAS system will be on the order of 10^−6^–10^−8^ at a laser power of 1 W. Using different novel approaches to address issues associated with background noises, PAS cell design, sensitivity of the detector, and signal processing methods, the detection limit can be effectively reduced to 10^−7^–10^−9^. Depending on different gas molecules, trace gases can be detected by the PAS technique at ppm – ppb levels.

**Figure 4.**
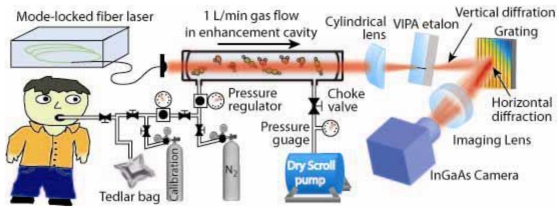
Schematic of the cavity-enhanced direct-frequency-comb spectrometer, along with the gas handling system for breath analysis (reproduced with permission from the Optical Society of America [50]).

Combining the new optical frequency comb technology with the CEAS technique, Ye *et al.* introduced OFC-CEAS [49], which combines high-sensitivity of the cavity enhancement technique with a broadband laser source, and demonstrated simultaneous detection of multiple breath species, as illustrated in Figure 4 [50]. The OFC-CEAS technique takes advantage of high peak intensities of the pulsed output to reach a wide spectral range from the UV to the far infrared via a nonlinear conversion process and creates a parallel detection scheme that records a large spectral bandwidth in a single laser shot. An initial OFC-CEAS system using a mode-locked fiber laser has demonstrated a minimum detectable absorption coefficient of 8 × 10^−10^ cm^−1^ (corresponding to an *A_min_* of 4 × 10^−8^ at a gas cell length of 50 cm) in a 200 nm spectral window in the NIR. Like the other forms of high-sensitivity laser spectroscopic techniques, the detection sensitivity of the OFC-CEAS can be further improved by 2–3 orders of magnitude when the operation of the frequency comb technology extends to the mid-infrared (MIR) spectral region.

## 4. Breath Biomarkers Detected by the Laser Spectroscopic Techniques

Of the 35 major breath biomarkers listed in Table 1, 14 biomarkers have been detected in actual human breath by the aforementioned laser spectroscopic techniques; some of the breath biomarkers have not been measured in actual breath gases to date, but have been measured in specially calibrated gas samples, which contain the biomarker of interest. The rest of the biomarkers have not been studied by the laser techniques so far. The spectral fingerprints of the 14 biomarkers range from the UV to the MIR. Selection of a spectral fingerprint for the detection of each breath species is based on an overall consideration of detection sensitivity, potential spectral interferences, availability of laser sources, and costs of potential breath sensors. Spectral fingerprints, laser techniques, detection limits, and corresponding literature references of each of the 14 biomarkers which have been detected with actual human breath by the laser techniques to date are listed in Table 2. It should be clearly noted that several biomarkers, such as butane (C_4_H_10_) [68], hydrogen peroxide (H_2_O_2_) [69], HCN [70,71], H_2_S [72-74], etc. listed in Table 1, which have been analyzed in non-breath gas samples by using laser-based techniques and can potentially be adopted for analysis of the biomarkers in actual breath, are not included in Table 2. Details of the breath analysis of these 14 biomarkers using the high-sensitivity laser techniques are described below in alphabetic order of their common chemical names.

### Acetaldehyde (C_2_H_4_O)

Acetaldehyde has been identified as a biomarker for alcoholism and lung cancer [18,112]. Normal human breath acetaldehyde ranges from 0 to 140 ppb [113]. Only one publication has reported the investigation of acetaldehyde in exhaled human breath [75], in which measurements of acetaldehyde in exhaled breath following the ingestion of 375 mL of wine (12.5% alcohol) were conducted by using the TDLAS technique. Acetaldehyde exhibits a strong absorption band in the spectral range of 5.49–5.75 μm (1,680 to 1,820 cm^−1^), which is attributed to the υ_4_ band, and the strongest absorption is at 1,764 cm^−1^. In that study, however, the absorption of acetaldehyde near 5.79 μm (1,727.1 cm^−1^) was measured at 26 Torr in an astigmatic Herriott cell with an effective path-length of 100 m. The detection limit for the acetaldehyde was reported to be 80 ppb and 30 ppb for an integration time of 5 and 45 s, respectively. The authors noted that if the molecular fingerprint at 5.67 μm (1,764 cm^-1^) is used, the detection limits can be improved by a factor of 2, which will be close to the required detection limit, 10 ppb, for analysis of acetaldehyde in human breath. Measurements of acetaldehyde in non-breath gas using the laser spectroscopic techniques have been reported and can be read elsewhere [114,115].

### Acetone ((CH_3_)_2_CO)

**Figure 5.**
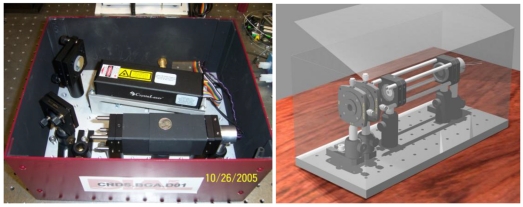
A prototype acetone breath analyzer using pulsed-CRDS at 266 nm. Left: The instrumental package; right: Detailed pictorial view of the optical cavity configuration.

The mean acetone concentration in healthy breath reported in the literature varies from 0.39 to 0.85 ppm, and the overall mean value is ∼0.49 ± 0.20 ppm with 1σ = 0.20 ppm [8,20,106]. Elevated acetone concentrations in the exhaled breath of diabetic subjects have been reported. Elevated breath acetone also exists in exhaled breath of children who are on a high-fat diet for the treatment of epilepsy [117]. Acetone concentrations in human breath can also be an indicator of congestive heart failure and cardiac index [118,119]. CRDS of acetone in the UV and NIR spectral regions have been reported. Wang *et al.* demonstrated a portable acetone detection device using CRDS at a single wavelength at 266 nm and evaluated the instrument performance using both standard acetone solution samples and actual human breath under various situations [76-78]. A photograph of the portable acetone detection device is shown in Figure 5.

Recently, a pilot-scale acetone breath analyzer has been developed and tested in both a research laboratory and a clinical study with 59 human subjects, including 34 Type 1 diabetic (T1D) patients, 10 Type 2 (T2D) diabetic patients, and 15 nondiabetic people [77]. The preliminary results include: (1) The mean acetone concentration in expired air of T1D subjects is 2.2 ppm. (2) Within the T1D group, the mean acetone concentration of juvenile-onset diabetic patients is significantly higher than that from adult-onset diabetes. (3) A linear correlation between the breath acetone and simultaneous BG level is observed and the correlation coefficient is R^2^ = 0.90 when all T1D subjects are grouped by blood glucose (BG) levels, 40–100, 101–150, 151–200, 201–419 mg per 100 mL. (4) A linear correlation between the mean group A1c and the mean group acetone concentration is also found with a correlation coefficient of R^2^ = 0.92 when the A1c of the T1D subjects are grouped by <7, 7–9.9, and 10–13. (5) Simultaneous measurements of BG and breath acetone of three juvenile-onset T1D subjects over a 24-hr time period show a correlation between the variations of the breath acetone and the BG levels. The observation of abnormal acetone concentrations in some T1D subjects, whose BG levels are well controlled (lower than 100 mg per 100 mL), indicates that mild ketosis is undiagnosed by measuring only BG. Analysis of breath acetone may be also achieved in the VUV spectral region by using synchrotron radiation. Regardless the cost issue, the spectral selectivity in the deep UV could be advantageous. Spectral fingerprints of acetone in the deep UV down to 115 nm have been reported recently by Ferreira da Silva *et al.* [120].

### Ammonia (NH_3_)

Ammonia concentration in normal human breath is in the range of 0.25–2.9 ppm. Ammonia is a biomarker of renal failure, Helicobacter pyroli and oral cavity disease [121,122]. One of the earliest measurements of ammonia in human breath was reported by Lachish *et al.* [79]. They used the TDLAS technique combined with a high resolution MIR lead-salt light source. In the near-real time measurement of ammonia, the laser was locked on the peak of the absorption line of ammonia at 10.74 μm (930.76 cm^−1^). The absorption of ammonia was observed in a sample cell with a path-length of 50 cm. The detection limit was 1 ppm with an integration time of 10 s. Manne *et al.* [81] reported a prototype breath gas analyzer based on the CRDS technique for quantification of ammonia in human breath. A thermo-electrically cooled distributed feedback (DFB) quantum cascade diode laser (QCDL) was used as the MIR laser source operating at 10.3 μm (970 cm^-1^). The system showed a detection limit of 50 ppb for ammonia with a response time of 20 s. Recently, the same group has developed a sensor for measurements of breath ammonia with a pulsed quantum cascade laser using intra- and inter-pulse spectroscopic techniques. The system utilized an astigmatic Herriott gas cell with a 150 m effective path-length and a pulsed DFB-QCDL operating at 10.6 μm. The detection limits for breath ammonia of the system were 3 ppb when the system was operated in the intra-pulse method with an integration time of less than 10 s and 4 ppb in the inter-pulse method with an integration time of ∼5 s [82]. Simultaneous measurements of multiple breath species, including ammonia, were demonstrated by the Ye group using the OFC-CEAS technique [50]. In that study, an NIR spectral window centered around 1.51 μm (6,623 cm^−1^) was used, where absorptions of ammonia and water were relatively overlapped. Using calibrated samples with 4.4 ppm ammonia in nitrogen as a reference, they obtained a detection limit of 18 ppb [50]. Moskalenko *et al.* reported an automated TDLAS system operating in the MIR and measured multiple compounds in smokers' and non-smokers' exhaled breath, including ammonia, carbon monoxide, methane, and carbon dioxide [83]. They reported ammonia concentrations in the smokers and non-smokers' breath had no significant difference and they were all in the range of 130–200 ppb. The detection limit for ammonia measured at 10.3 μm was 5 ppb for a response time of 30 sec.

### Carbon dioxide (CO_2_) and ^13^C-isotope

Carbon dioxide and carbon isotopes have been established as a biomarker of Helicobacter pyroli infections, liver malfunction, and excessive growth of bacteria in body etc. [123-127]. CO_2_ has a strong absorption around 4.3 μm (2,326 cm^−1^) which allows highly sensitive detection of CO_2_ and its ^13^C isotope using direct laser absorption spectroscopy with MIR laser sources, such as lead-salt lasers as shown by Becker *et al.* [128] and DFG light sources as shown by Erdelyi *et al.* [129]. Crosson *et al.* [85] conducted measurements of CO_2_ in breath samples using cw-CRDS with an external cavity diode laser (ECDL) at 1.597 μm (6,262 cm^−1^) in a laboratory system at 100 Torr, a detection limit of 3 ppm for CO_2_ was demonstrated.

Analysis of carbon isotopes can be used for drug administration, and current breath analysis of ^13^CO_2_/^12^CO_2_ isotopic ratio is predominantly by a GC-MS method. While two breath carbon isotope studies using high-sensitivity laser spectroscopic techniques have been reported, several laser spectroscopic measurements of ^13^C isotope have been done with non-breath gases. Crosson *et al.* demonstrated the determination of ^13^C isotopic abundance in ^12^CO_2_ mixed with nitrogen (5% CO_2_ in N_2_) using a cw-CRDS system at reduced pressures. The measurement accuracy of the ^13^C isotopic ratio was as high as 0.22*‰* [85]. Note that isotopic abundance ratio, e.g., ^13^C isotopic abundance δ^13^C is typically expressed relative to the PDB standard by the common notation:

δC13=Rsample−RstandardRstandard×1,000

where *R* = ^13^C/^12^C, the unit of δ^13^C is parts per mil (*‰*). The international PDB standard of *R_standard_* is 0.0112372. The similar notation is used to define other isotopic abundance ratios, such as δ^18^O, δ^18^H, etc. Thorpe *et al.* reported measurements of breath carbon (^13^C) and oxygen (^18^O) isotopic ratios using the OFC-CEAS technique and they found the carbon (^13^C) and oxygen (^18^O) isotopic ratios in the breath samples were δ^18^O = −9.1 ± 4.2*‰* and δ^13^C = −28.8 ± 4.1*‰*, respectively [50]. Erdelyi and co-workers measured ^13^CO_2_/^12^CO_2_ isotopic ratio in volcanic gas emissions using direction absorption spectroscopy with a dual gas cell scheme, and they demonstrated a measurement accuracy of 0.8*‰* at 4.35 μm in a field-deployable gas sensor [129]. Castrillo and co-workers reported isotopic analysis of ^13^CO_2_/^12^CO_2_ using direct laser absorption spectroscopy with a DFB diode laser operating at 2.008 μm and whose optical components were built on a 60 cm × 60 cm breadboard. Four pure CO_2_ samples with known δ^13^C values were measured at a low pressure of 13.5 Torr. The measurement accuracy of δ^13^C was ∼0.5*‰* [130,131]. More recently, Wahl *et al.* have combined NIR cw-CRDS with a sample pre-concentration method that is usually employed in sample preparations for GC-MS and demonstrated measurement of δ^13^C in atmosphere with a measurement accuracy of 0.2*‰* [132]. Apart from these, Horner *et al.* measured CO_2_ using the TDLAS technique at 1.6 μm and reported a precision of 1*‰* [133]. Nelson *et al.* have reported the measurements of ^13^C in CO_2_ using a pulsed quantum cascade laser operating at 4.3 μm (2,326 cm^−1^); they achieved 0.2*‰* precision [134]. Recently, Dirk *et al.* reported measurements of ^13^C in CO_2_ at 4.32 μm (2,315 cm^−1^) by using a DFG laser system; they obtained a precision of 0.05*‰* [135].

### Carbon monoxide (CO)

Carbon monoxide is a biomarker of hyperbilirubina, oxidative stress, respiratory infections, and asthma [136-140]. CO is also used to monitor bilirubin production in smoking cessation and access the lung diffusion capacity. Moeskops *et al.* conducted measurements of CO in human breath at 4.6 μm (2,176 cm,^-1^) using the TDLAS technique with a thermo-electrically cooled DFB-QCDL [141]. A detection limit of breath CO of 175 ppb for a 0.2 s integration time was reported. Breath CO was also measured in smokers' breath samples in the Ye group by using the OFC-CEAS technique, and they found that the average CO in exhaled smokers' breath was 6.5 ppm (1,538 cm^−1^), which was five times higher than the 1.3 ppm measured in non-smokers' breath [49]. A detection limit of 900 ppb for CO at 1.564 μm (6,394 cm^−1^) was reported. In the early work reported by Moskalenko *et al.* [83], CO and CO_2_ were simultaneously measured in the exhaled breath of smokers and non-smokers by a TDLAS system operating at 5.0 μm (2,000 cm^−1^). CO concentrations in the exhaled breath of the smokers and non-smokers were in the ranges of 2–20 ppm and 0.4–0.8 ppm, respectively. A detection limit for CO of 50 ppb was reported.

### Carbonyl sulfide (OCS)

Carbonyl sulfide (OCS) has been identified as a biomarker of liver-related diseases [142]. Elevated concentrations of OCS have been observed in patients who have undergone lung transplantation and have had acute allograft rejection [143]. The production of exhaled OCS is attributed to oxidative metabolic processes of carbon disulfide and the incomplete metabolism of methionine. A few measurements of breath OCS using laser spectroscopic techniques have been reported to date. Halmer *et al.* [46] reported the determination of concentrations of OCS present in both human breath and the atmosphere. They employed the CALOS technique in the MIR region with a cw-CO_2_ laser operating at 4.9 μm (2,041 cm^−1^). The detection limits achieved were 7 ppt and 9 ppt in the ambient air and breath gas, respectively. Wysocki and co-workers constructed a compact OCS sensor using a Herriott cell which had an effective path-length of 36 m [87]. Trace OCS gas in exhaled human breath was measured in the spectral range of 4.85–4.87 μm (2,054.5–2,060.5 cm^−1^). The gas cell pressure was maintained at 60 Torr, so that the transition lines of OCS and CO_2_ in that spectral region were resolved. A detection limit of 1.2 ppb was achieved at 4.86 μm (2,058 cm^−1^) for a 0.4 s acquisition time. Roller *et al.* demonstrated quantification of trace OCS using a TDLAS-based compact absorption spectrometer with a thermo-electrically cooled MIR tunable QCDL at 4.86 μm [90], the potential of being used as a breath analyzer in measuring OCS in exhaled breath was demonstrated. A 30 ppb detection limit for OCS was reported. They also reported a high-selectivity of the system capable of resolving two stable isotopes of OCS: ^16^O^12^C ^32^S and ^16^O^12^C ^34^S. Apart from the OCS measurements in breath gas, Fried *et al.* conducted experiments to measure OCS in ambient air [144]. They used the TDLAS technique combined with a cryogenically cooled cw lead-salt laser. A detection limit of 7 ppt was reported. Fischer *et al.* measured trace OCS using the PAS technique with a difference frequency generation (DFG) laser system at 3.14 μm (3,185 cm^−1^) [145]. The detection limit of OCS was reported to be 40 ppm.

### Ethane (C_2_H_6_)

**Figure 6.**
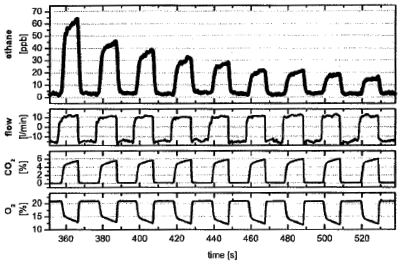
Typical measurement of single-breath concentrations of C_2_H_6_, CO_2_, and O_2_. Each single expiration is analyzed separately. Only 160 s are displayed in the 360–520 s time windown. The complete measurement took 42 min. (Reproduced with permission from Spinger Link [45]).

In addition to being biomarkers of asthma, lipid peroxidation, and vitamin E deficiency as previously mentioned [55-58], ethane is also an indicator of diseases like sceloderma and cystic fibrosis [146,147]. Ethane concentration in human breath has been reported to be in the range of 0–12 ppb [94]. A series of experiments have been conducted to measure breath ethane concentrations using different laser spectroscopic techniques. Measurements of ethane in the MIR was mostly conducted around 3.3 μm (3,030 cm^−1^). Dahnke *et al.* reported for the first time real-time monitoring of ethane in human breath with CALOS using a CO overtone laser operating in the range of 2.6–4.0 μm (2,500–3,846 cm^−1^). The measurements were performed in a gas cell at 76 Torr and a detection limit of 100 ppt was reported. The measuring time was 5 s. The study demonstrates the capability of the CALOS technique for fast and sensitive analysis of fractional ethane in exhaled human breath [43]. Figure 6 shows the recent measurement results of breath ethane, CO_2_, and O_2_ using the CALOS technique [45].

Halmer *et al.* demonstrated a transportable MIR laser CALOS spectrometer for online monitoring of trace ethane in exhaled breath. A periodically poled lithium niobate (PPLN) crystal pumped by an Nd:YAG laser and a diode laser with tapered amplifier were used to construct a DFG laser source at 3.3 μm (3,030 cm^−1^) [94]. The pressure inside the cavity was kept at 56 Torr. The reported detection limit for breath ethane was 270 ppt with a time resolution of 1 s. Parameswaran *et al.* reported a simple, compact system for online monitoring of ethane in human breath using the ICOS technique [92]. A DFB interband cascade laser operating under liquid nitrogen cooling (77 K) generated a laser beam at 3.34 μm (2,994 cm^−1^). The pressure inside the cell was 130 Torr and the minimum detectable ethane concentration was 0.12 ppb/(Hz)^1/2^. Skeldon and co-workers reported the application of the TDLAS technique in the measurements of exhaled ethane in patients with lung cancer [93]. Breath samples from 52 patients were collected, and a simple tunable diode laser spectrometer with a lead-salt laser at 3.4 μm (2,941 cm^−1^) was used to analyze ethane in breath samples. The measurements were compared with the ones using GC-MS. A detection limit of 0.1 ppb was reported. Fischer *et al.* constructed a compact DFG diode laser combined with the PAS technique for measuring the concentration of trace ethane [145]. Measurements were performed with the sample consisting of 9 ppm methane and 10 ppm ethane diluted in synthetic air at a total pressure 760 Torr. A detection limit for ethane was reported to be 1.8 ppm.

### Ethylene (C_2_H_4_)

Ethylene, also known as ethene, is a biomarker for ultra violet radiation damage of human skin and lipid peroxidation in the lung epithelium [61,148,149]. Analysis of ethylene in exhaled breath using a laser spectroscopic technique has only been reported to date by Dumitras *et al.* [97] They implemented the PAS technique with a CO_2_ laser source to measure breath ethylene in a smoker and a non-smoker. A potassium hydroxide (KOH) trap was employed to trap breath CO_2_ to minimize the absorption interference, thus improve detection sensitivity. They achieved a 17 ppb detection limit for breath ethylene by using an 88 cm^3^ volume KOH trap. Puiu *et al.* have also demonstrated a PAS based sensor for the online monitoring of ethylene [95].

### Formaldehyde (HCHO)

Formaldehyde has been found to be a biomarker of lung cancer and breast cancer [150]. Laser spectroscopic techniques have proven to be very useful in detecting formaldehyde in concentrations as low as ppb levels. Formaldehyde has a strong absorption around 3.53 μm (2,833 cm^−1^). Rehle *et al.* reported the detection and quantification of atmospheric formaldehyde using the TDLAS technique with a DFG laser source operating at 3.53 μm (2,833 cm^−1^) [99]. The sensor was reported to have excellent time resolution (on the order of seconds) and continuously operated unattended for a long period of time. The multipass gas cell used in that work had a 100 m effective path-length and constant pressure of 40 Torr. The detection limit for formaldehyde with this system was 0.32 ppb. The spectroscopic method was evaluated by a conventional wet chemical technique and the results were in agreement with each other. Richter and co-workers demonstrated the development of a TDLAS-based compact tunable DFG laser system for the quantification of airborne trace gases including formaldehyde [103]. The gas samples were contained in an astigmatic Herriott gas cell at a cell pressure of 40 Torr. The minimum detectable concentration for formaldehyde was reported to be as low as 77 ppt for an integration time of 1 min. The Murtz group demonstrated real-time detection of formaldehyde present in ambient air using the CALOS technique [100]. The experimental setup consisted of a tunable CO overtone sideband laser operating at 3.5 μm (2,850 cm^−1^). The experiment was conducted at 23 Torr, and a detection limit of 2 ppb was reported. Angelmahr *et al.* reported a laboratory-based system using the PAS technique with an OPO laser source for selective and sensitive detection of formaldehyde in nitrogen [101]. The system demonstrated the detection of formaldehyde in laboratory air without using pre-concentration processes. The measurements were conducted at the ν_1_ absorption line located at 3.5 μm (2,857 cm^−1^). A detection limit of 3 ppb was reported for an integration time of 3 min. Miller *et al.* reported the detection and measurement of formaldehyde using the ICOS technique combined with a cw-DFG interband cascade laser at 3.53 μm (2,833 cm^−1^) [98]. A detection limit of 150 ppb for formaldehyde for a 3 s integration time was reported. Recently, Ciaffoni *et al.* performed proof-of-principle experiments to measure atmospheric formaldehyde at 3.5 μm (2,857 cm^−1^) using the TDLAS-WM technique [104]. They reported a detection limit of 1.2 ppm. Very recently, Horstjann *et al.* have measured formaldehyde using the QEPAS technique with a cw-DFG interband cascade laser, and a detection limit of 0.6 ppm for a 10 s integration time has been reported [102].

### D/H isotope

D/H isotopic ratio measurements in exhaled breath can be of significant importance. The ratio can provide information about glucose and cholesterol synthesis rates inside the body [151]. Measurements of dilutions of D_2_O can be used to determine the total body water (H_2_O) [152]. Recently, Bartlome *et al.* have demonstrated breath analysis of the D/H isotope ratios using a home-built tunable, pulsed DFG laser source operating in the wavelength range of 3.5–3.65 μm (2,740–2,857 cm^−1^) combined with a high temperature multipass gas cell [91]. The minimum detectable absorption coefficient of the system was 2.6 × 10^−6^ (3σ). Repetitive measurements of the deuterium isotopic abundance ratio, δ^2^H, were carried out before intake of D_2_O, and the measurement precision was 23*‰* (1σ). The temporal behavior of δ^2^H after D_2_O intake was also monitored. They demonstrated the capability of continuous monitoring of the increase of δ^2^H in exhaled water vapor after oral ingestion of a clinically relevant amount of D_2_O.

### Methane (CH_4_)

Methane has been identified as a biomarker for colonic fermentation and intestinal problems [153]. Methane can be measured based on its NIR and MIR spectral fingerprints. Several experiments using different laser spectroscopic techniques have been conducted around 3.3 μm (3,030 cm^−1^) and 7.8 μm (1,282 cm^−1^). Moskalenko *et al.* reported the analysis of methane in human breath using the TDLAS technique [83]. In that study, the methane concentratons in a non-smoker and a smoker's breath gases were compared and the time variations of methane concentrations in the two subjects were investigated. The absorption line of methane, belonging to the ν_3_ fundamental band at 3.47 μm (2,882 cm^−1^), was used for the detection of methane. The experimental setup used an InSbAs diode laser source and a single-path cell of 2 m. The measurements were conducted at 76 Torr. A detection limit of 0.5 ppm for methane was reported, which was sufficiently sensitive to detect methane in normal human breath, which has in average concentration of 3–8 ppm. The variation in methane concentration in the exhaled breath with time was also investigated. Scotoni *et al.* developed a system for simultaneous detection of multiple gases including methane. The system employed a resonant PAS technique with an ECDL with approximately 40 nm single-mode coarse tunability [154]. The photoacoustic cell had a length of 60 mm and a diameter of 2 mm. The experiment was conducted at 1.63 μm (6,115 cm^−1^). A data fitting procedure was developed and employed to limit the need of a reference cell and improve the detection sensitivity. A detection limit of 6 ppm for CH_4_ was obtained with a 0.3 mW laser power. Measurements of trace methane in other than breath gases by several other groups can be seen elsewhere [145,155-160].

### Methylated amines (MAs)

Variations in concentrations of methylated amines in human urine, sweat, and breath are associated with various diseases or altered metabolism in the body. Marinov *et al.* conducted experiments to measure concentrations of methylated amines in a synthetic gas mixture [105]. A cavity ringdown spectrometer was developed to measure the trace mono-methylamine (MMA, CH_3_NH_2_) and dimethylamine [DMA, (CH_3_)_2_NH]. The first overtone of the NH stretch vibration around 1.52 μm (6,579 cm^−1^) was chosen as the spectral fingerprint, where both MMA and DMA showed modest absorption at 1.52 μm. The minimum detectable absorption coefficient was 1.55 × 10^-8^ cm^−1^ (1σ), corresponding to detection limits of 350 ppb and 1.6 ppm for MMA and DMA in synthetic non-absorbing mixtures, respectively. These detection limits are equivalent to 10 ppm and 60 ppm for MMA and DMA, respectively, in exhaled human breath.

### Nitric oxide (NO)

Along with being a well-established biomarker for diseases like asthma, hypertension, and rhinitis [51-54], nitric oxide (NO) is also considered as non-invasive biomarker of air way inflammation like lipopolysaccharide-induced airway inflammation, and lung inflammation [161-163]. Analysis of exhaled NO has been used to monitor asthma treatment using inhaled corticosteroids. NO is among the very few biomarkers which have been detected by most of the available laser spectroscopic techniques. NO has a strong absorption band around 5 μm (2,000 cm^−1^). Several publications show that NO can be detected by using laser absorption techniques with detection sensitivities up to the ppb levels. Roller *et al.* carried out real-time monitoring of breath NO in human subjects [109]. The method used an internal calibration through simultaneously measuring concentrations of NO and CO_2_. This approach eliminated the need to calibrate the system with certified standard gases. The experiment was conducted with a TDLAS system in conjunction with a lead-salt laser operating near 5.2 μm (1,923 cm^−1^). Breath samples were collected in disposable mouth pieces. A multipass Herriott cell with a volume of 16 L was used as the sample cell in the experiment. The detection limit obtained for NO was 1.5 ppb for a 4 s integration time. The effect of sample cell pressure, exhalation times, and ambient NO concentration on the measurements of the breath NO were also studied. The system demonstrated suitableness for clinical application [109]. McCurdy *et al.* reported a laboratory-scale ICOS-based NO/CO_2_ sensor, capable of performing real-time measurements of NO and CO_2_ in a single breath cycle [88]. The system employed a cw-DFB quantum cascade laser operating at 5.22 μm (1,915 cm^−1^) and a high finesse cavity with a cell length of 50 cm and sample volume of 60 mL. The instrument was capable of measuring CO_2_ and NO in exhaled human breath simultaneously. Prior to the analysis, the ICOS sensor was calibrated with a standard reference mixture of 76 ppb NO in N_2_. The sample flow rate into the ICOS system was kept fixed at 300 mL/min. With human breath samples, a detection limit of less than 1 ppb in a 4 s integration time at 100 Torr was reported. Additionally, the performance of ICOS sensor was compared with a commercially available chemiluminescent NO analyzer. The sensor showed the capability of detecting multiple clinically important VOC's. Namjou *et al.* demonstrated measurements of NO in the MIR in a diverse sample population using an experimental setup based on the TDLAS technique [110]. The experiment was conducted at 5.22 μm (1,916 cm^−1^) with a cryostat-housed lead-salt tunable semiconductor laser source. The system used a 0.3 L astigmatic Herriott cell with an optical path-length of 36 m. The gas pressure inside the cell was maintained at 45 Torr. Breath samples were collected from 799 human subjects with their ages ranging from 5 to 64. The absolute values of NO were quantified using NO/CO_2_ absorption ratios. The concentration of CO_2_ was measured by a non-dispersive infrared CO_2_ sensor, which was coupled with the TDLAS system. The minimum detectable concentration for NO was reported to be 2 ppb. This technique was shown to be free from the regular routine calibration using standard cylinder gases. Recently, Heinrich and co-workers have reported online measurements of ^15^NO/^14^NO in nasal air using a newly constructed CALOS-based spectrometer [111]. The experiment was performed at 5.3 μm (1887 cm^−1^). The leak out signal was detected with a liquid nitrogen cooled InSb photodetector. The pressure was set at 53 Torr. The minimum detectable concentration was 7 ppt for an integration time of 70 s. Bakhirkin *et al.* demonstrated the potential of an ICOS spectrometer for the detection and measurement of NO in the exhaled breath [39]. The spectrometer implemented a thermo-electrically cooled pulsed QCDL as the laser source and a high finesse ringdown cavity that had two highly reflective mirrors (R > 99.97%) separated 50 cm, generating an effective optical path-length of 1.5 km. The system showed the capability of measuring both NO and CO_2_ simultaneously. The study achieved a detection limit for NO in breath samples of <1 ppbv with a 4 s integration time. Menzel *et al.* demonstrated a system based on the CEAS technique for measuring atmospheric NO, and the potential of the system for biomedical application was investigated [107]. The system utilized a cw-QCDL laser operating at 5.2 μm (1,923 cm^−1^) under cryogenic conditions. With a cavity length of 35.5 cm, equivalent to an effective path-length of 670 m, they obtained a detection limit of 16 ppb with an integration time of 200 s. The same group used the same QCDL system, operating at the liquid nitrogen temperature and 50 Torr, with an effective optical path-length of 100 m, they achieved a detection limit for NO of 3 ppb. Moeskops *et al.* explored the capability of using a single mode thermo-electrically cooled cw- QCDL to detect and measure NO in sub ppb levels [141]. The thermo-electrically cooled cw-QCDL operating between 5.41–5.39 μm (1,847 and 1,854 cm^−1^) was combined with wavelength modulation spectroscopy and an astigmatic multipass cell with an effective optical path of 76 m was used. They achieved a detection limit of 0.2 ppb for NO in N_2_ with a 30 s integration time at 76 Torr and 5.4 μm. Kosterev *et al.* reported measurements of NO in N_2_ using the CRDS technique with a cw-QCDL operating at 5.2 μm (1,923 cm^−1^) [108]. They obtained a detection limit of 0.7 ppb for an integration time of 8 s. This detection limit reached the detection limit of 1 ppb, suggested by the American Thoracic Society, yet the response time was still longer than the suggested value, 0.5 s.

## 5. Current Status of Prototype and commercialized Breath Sensors

Breathmeters for the detection of several breath biomarkers are commercially available from Ekips Technologies Inc. [164] and their clinical applications are expected to be approved by the U.S. Food and Drug Administration (FDA). Current breathmeters, which are based on the TDLAS technique using a lead-salt laser source in the MIR, are able to detect NO, CO_2_ carbon isotope ratio, NH_3_, etc., biomarkers. Using the PAS technique, an array of breath sensors developed by Pranalytica Inc. is commercially available for human breath analysis [165]. Breath biomarkers including NH_3_, CH_4_, H_2_S, etc. can be detected with detection limits of ppb – ppt levels. Using the original form of the CRDS technique with a palm-size, single-mode, pulsed 266-nm laser source, a prototype acetone breath analyzer has been tested with healthy people and diabetic outpatients in a clinical study [76-78]. This prototype acetone breath analyzer, which has not yet been commercialized, is available for clinical testing and data collection associated with breath acetone. In addition to the breath gas sensors/analyzers that have been tested in clinics, several laser spectroscopic gas sensors, as discussed in the preceding section, have been tested with online and/or offline breath analysis of the 14 breath biomarkers listed in Table 2. For example, a cw-CRDS spectrometer has been demonstrated for measurements of ^13^CO_2_/^12^CO_2_ in offline breath samples; standalone gas sensors based on ICOS and PAS using a QCDL have been demonstrated for measurements of trace acetone, formaldehyde, nitric oxide, etc. [166]. These sensors are ready for breath analysis in clinics.

## 6. Challenges and Perspectives

Challenges in breath analysis using laser spectroscopic techniques may include four major aspects: costs of future POC breath analyzers, spectral interferences, breath sample maneuver, and quantitative correlation of the concentration of a biomarker with the level of a blood-test-based diagnostic agent. The telecommunications diode lasers are inexpensive; however, they restrict spectroscopic measurements to be conducted in the NIR spectral region, where line strengths of molecular overtone transitions are several orders of magnitude weaker than that of electronic transitions in the UV and fundamental bands in the MIR. On the other hand, the current UV and MIR laser sources are expensive and bulky, which are not suitable for a POC instrument. For instance, bulky lead-salt lasers, DFG lasers, or OPO systems, operating in the MIR are only practically useful for laboratory breath analysis. QCDL technology has gained rapid advancement during the last ten years. They can operate in non-cryogenic conditions; and size and cost of QCDL systems have been significantly reduced. Nevertheless, the current cost of a breath sensor using a QCDL is still beyond consideration for a home-owned medical care instrument.

The interference issue is two-fold: spectroscopic interferences and bio-interferences. The former depends on the selection of detection fingerprints for a biomarker. Regardless of instrument costs, using spectral fingerprints in the NIR would be inferior to the UV or MIR, due to the abundant NIR absorption of water vapor in breath and the interfering absorption of the first overtone of C-H and other fundamental vibrations. A careful selection of an NIR spectral fingerprint which does not overlap with an absorption of other molecules is achievable, but often sacrifices the detection sensitivity. The latter is determined by the nature of each individual biomarker and its associated physiological symptoms. For instance, acetone is a biomarker for T1D; however, an epileptic patient on a high-fat ketogenic diet also has elevated breath acetone. In this case, more than one breath biomarker or a relative abundance pattern of several breath biomarkers may be developed as a breath fingerprint for a particular disease or metabolic disorder.

The breath gas maneuver is a challenge especially for the laser-based breath analysis since the laser-based breath analysis is performed via on-line, real-time breath gas introduction to the gas cell. Single exhaled air or multiple exhaled airs contain the dead-space air and alveolar air. Concentrations of VOCs coming from the oral cavity or being generated in the airways are in the dead-space air than in the alveolar air. Due to dilution in the dead-space, concentrations of endogenous blood-borne VOCs in mixed expiratory samples that are often used in breath analysis are lower than those in alveolar air. The American Thoracic Society recommends for a standarized procedure for the online measurements of exhaled NO in adults using a respiratory maneuver to separate nasal contamination from those of lower air origin [167]. How to handle the breath sampling (without separation of the dead space air and the alveolar air) for on-line, real-time, clinical breath analysis is a challenge even though a laser technique is ready for sensitive and selective measurements of a breath biomarker.

Finally, the fourth challenge is not only related to breath analysis using the laser-based techniques, but is also relevant to breath analysis in general. Current research efforts in breath analysis are mainly focused on technology development and initial breath testing. A large scientific gap exists between a quantified concentration of a breath biomarker and the level of an established blood-test-based diagnostic agent. For example, we may obtain an accurate, near-real time, breath acetone concentration in a T1D patient using the CRDS-based acetone breath analyzer, but we still do not know quantitatively the blood glucose level of the patient from the breath acetone concentration because of the lack of a quantitative correlation of breath acetone concentration with the blood glucose level. This knowledge gap makes the breath acetone measurements function as only a monitoring means, instead of a diagnostics means for diabetic patients.

Breath analysis for clinical use is in its infancy. Breath analysis using laser spectroscopic techniques is only a very recent advancement. To some extent, the advancement takes a ride on modern telecommunications industries, which create a new generation of diode lasers capable of covering a wide spectral range of molecular fingerprints. Although current advanced laser sources, such as QCDLs, are still too expensive for a home-owned breath sensor, a laser-based breath sensor has already proven to be significantly less expensive than an MS-based system. Additionally, modern coating technologies make high reflectivity mirrors technically and economically more available; thus, costs of constructing a high-finesse optical cavity for the CRDS, ICOS, and OFC-CEAS techniques are expected to be reduced significantly. These advancements will further accelerate the movement of the laser-based breath analysis from laboratory research to initial clinical testing. Some laser-based breath sensors are commercially available. However, it is worth being emphasized that laser-based breath analysis is a new interdisciplinary field, which involves with laser and optical engineering, physics and chemistry, and medicine. Moving breath analysis forward requires effective communication and a broad collaboration among spectroscopists, optical engineers, and clinicians. While the MS-based techniques will continue to play an indispensable role in the identification and quantification of new breath biomarkers, laser-based breath analysis will play its unique role in POC, real-time, at-home, noninvasive disease diagnosis and metabolic status monitoring.

## 7. Summary

This article gives an overview on the new field of breath analysis using the high-sensitivity laser spectroscopic techniques: TDLAS, CRDS, ICOS, CEAS, CALOS, PAS, QEPAS, and OFC-CEAS. Of the 35 major established biomarkers, 14 breath biomarkers, their associated physiological symptoms, the spectral fingerprints in the UV – MIR and laser detection techniques used, and the detection sensitivities achieved in actual human breath analysis have been updated. Challenges and perspectives of laser-based breath analysis are discussed.

## Figures and Tables

**Table 1.** 35 established biomarkers and their physiological symptoms.^a^

**Biomarkers**	**Metabolic Disorders / Diseases**
Acetone (OC(CH_3_)_2_)	Lung cancer, diabetes, dietary fat losses, congestive heart failure, brain seizure
Acetaldehyde (CH_3_CHO)	Alcoholism, liver related diseases, lung cancer
Ammonia (NH_3_)	Renal diseases, asthma
Butane (C_4_H_10_)	Tumor marker in lung cancer
Carbon monoxide (CO)	Oxidative stress, respiratory infection, anaemias
Carbon disulphide (CS_2_)	Schizophrenia, coronary, and artery diseases
Carbon dioxide (CO_2_) (^13^C-Isotopes)	Oxidative stress
Carbonyl sulfide (OCS)	Liver related diseases
Ethane (C_2_H_6_)	Vitamin E deficiency in children, lipid peroxidation, oxidative stress
Ethanol (C_2_H_5_OH)	Production of gut bacteria
Ethylene (C_2_H_4_)	Lipid peroxidation, ultra violet radiation damage of skin
Hydrogen (H_2_)	Indigestion in infants, intestinal upset, colonic fermentation
H/D isotope	Body water
Hydrogen peroxide (H_2_O_2_)	Asthma
Hydrogen cyanide (HCN)	Pseudomonas aeruginosa in children affected with cystic fibrosis
8-Isoprostane	Oxidative stress
Isoprene	Blood cholesterol
Methane (CH_4_)	Intestinal problems, colonic fermentation
Methanethiol (CH_3_SH)	Halitosis
Methanol (CH_3_OH)	Nervous system disorder
Methylated amines	Protein metabolism in body
Methyl nitrate (CH_3_NO_3_)	Hyperglycemia in Type 1 diabetes
Nitrogen monoxide (NO)	Asthma, bronchiectasis, hypertension, rhinitis, lung diseases
Nitrotyrosine (C_9_H_10_N_2_O_5_)	Asthma
Oxygen (O_2_)	Respiration
Pentane (C_5_H_12_)	Peroxidation of lipids, liver diseases, schizophrenia, breast cancer, rheumatoid arthritis
Pyridine (C_5_H_5_N)	Periodontal disease
Sulfur compounds	Hepatic diseases and malordor, lung cancer
Hydrocarbons (Toulene (C_6_H_5_CH_3_), Benzene (C_6_H_6_), Heptane (C_7_H_16_), Decane (C_10_H_22_), Styrene (C_8_H_8_), Octane (C_8_H_18_), Pentamethylheptane (C_12_H_26_))	Lipid peroxidation, lung cancer, oxidative stress, airway inflammation

a Note that except for NO, the only one that has been approved by the U.S. Food and Drug Administration as a biomarker of chronic airway inflammation in asthma, other breath compounds listed in Table 1 should be more accurately termed as “potential biomarkers” while the term “biomarker(s)” is used throughout the paper.

**Table 2.** Spectral fingerprints, laser spectroscopic techniques used, and detection limits achieved for the 14 major biomarkers, which have been analyzed with actual human breath by the laser spectroscopic techniques. The numbers in the parenthesis in the Column 4 indicate the measuring times in which the corresponding detection limits were achieved.

**Breath bio- markers (chemical formula)**	**Spectral fingerprints (UV-MIR) (μm)**	**Laser spectroscopic technique**	**Detection limits/Measured concentrations**	**References**
Acetaldehyde (CH_3_COH)	5.79	TDLAS	80 ppb (5 sec)	Kamat *et al.* [75]
Acetone (OC(CH_3_)_2_)	0.266	CRDS	0.2 ppm	Wang *et al.* [76-78]
Ammonia (NH_3_)	9–10.7	PAS	100 ppm (3 sec)	Narasimhan *et al.* [79]
NH_3_	11.0	TDLAS	1 ppm (10 sec)	Lachish *et al.* [80]
NH_3_	10.0	TDLAS	50 ppb (20 sec)	Manne *et al.* [81]
NH_3_	10.0	TDLAS	3 ppb (10 sec) 4 ppb (5 sec)	Manne *et al.* [82]
NH_3_	10.3	TDLAS	5 ppb (30 sec)	Moskalenko *et al.* [83]
NH_3_	1.5	OFC-CEAS	4 ppm	Thorpe *et al.* [50]
Carbon dioxide and C-isotope [CO_2_ & ^13^CO_2_/^12^CO_2_]	4.23	PAS	7 ppb	Herpen *et al.* (CO_2_ from insects in atmosphere) [84]
CO_2_	1.6	CRDS	3 ppm	Crosson *et al.* [85]
CO_2_	1.59	TDLAS/WM	100 ppm	Weldon *et al.* [86]
CO_2_	4.9	TDLAS	0.5 ppm (50 μs−1 ms)	Moskalenko *et al.* [83]
CO_2_	4.8	TDLAS	∼5.1% as compared with a spectrum of 5% CO_2_ in air.	Wysocki *et al.* [87]
CO_2_	4.9	CALOS	(3.778 ± 0.004)%	Halmer *et al.* [46]
CO_2_	5.2	ICOS	-	McCurdy *et al.* [88]
^13^CO_2_/^12^CO_2_	1.6	CRDS	Precision, 0.2*‰*	Crosson *et al.* [85]
^13^CO_2_/^12^CO_2_	1.6	OFC-CEAS	Precision, 4.1*‰*	Thorpe *et al.* [50]
Carbon monoxide (CO)	1.6	OFC-CEAS	900 ppb	Thorpe *et al.* [50]
CO	4.6	TDLAS	0.5 ppm	Moskalenko *et al.* [83]
CO	4.88	TDLAS	-	Lee *et al.* [89]
Carbonyl sulphide (OCS)	4.86	TDLAS	1.2 ppb	Wysocki *et al.* [87]
OCS	4.86	TDLAS with Thermo- electrically QC laser	30 ppb	Roller *et al.* [90]
OCS	4.9	CALOS	438 ppt	Halmer *et al.* [88]
D/H isotopic ratio (D_2_O/H_2_O)	3.50-3.65	TDLAS	55.2% ± 1.8% body water	Bartlome *et al.* [91]
Ethane (C_2_H_6_)	3.4	OA-ICOS	0.12 ppb	Parameswaran *et al.* [92]
C_2_H_6_	3.4	TDLAS	0.1 ppb	Skeldon *et al.* [93]
C_2_H_6_	3.3	CALOS	270 ppt	Halmer *et al.* [45]
C_2_H_6_	3.0	CALOS	100 ppt	Dahnke *et al.* [43]
C_2_H_6_	2.6–4.0	CALOS	500 ppt (<800 ms)	von Basum *et al.* [44]
C_2_H_6_	3.4	TDLAS	0–12 ppb	Patterson *et al.* [94]
C_2_H_6_	3.3	PAS	-	Puiu *et al.* [95]
C_2_H_6_	3.4	TDLAS/WM	70 ppt	Skeldon *et al.* [96]
Ethylene (C_2_H_4_)	10.5	PAS	-	Puiu *et al.* [95]
C_2_H_4_	9.2–10.8	PAS	-	Dumitras *et al.* [97]
Formaldehyde (CH_2_O)	3.53	ICOS	150 ppb	Miller *et al.* [98]
CH_2_O	3.53	TDLAS	320 ppt	Rehle *et al.* [99]
CH_2_O	3.53	CALOS	2 ppb	Dahnke *et al.* [100]
CH_2_O	3.53	PAS	3 ppb	Angelmahr *et al.* [101]
CH_2_O	3.53	QEPAS	0.6 ppm (10 sec)	Horastjann *et al.* [102]
CH_2_O	3.53	TDLAS	77 ppt (1 min)	Richter *et al.* [103]
CH_2_O	3.53	TDLAS	1.2 ppm	Ciaffoni *et al.* [104]
Methane (CH_4_)	3.35	TDLAS	0.5 ppm (50 μs−1 ms)	Moskalenko *et al.* [83]
Methylamine (CH_3_NH_2_)	1.51–1.53	CRDS	2.3 ppm	Marinov *et al.* [105]
Dimethylamine (CH_3_)_2_NH_3_)	1.51–1.53	CRDS	10 ppm	Marinov *et al.* [105]
Nitric Oxide (NO)	5.2	ICOS	1 ppb (4 sec)	Silva *et al.* [39]
NO	5.2	TDLAS	2 ppb	Namjou *et al.* [106]
NO	5.2	TDLAS	3 ppb (200 sec)	Menzel *et al.* [107]
NO	5.2	CEAS	16 ppb	Menzel *et al.* [107]
NO	5.2	CRDS	0.7 ppb	Kosterev *et al.* [108]
NO	5.2	TDLAS	1.5 ppb (4 sec)	Roller *et al.* [109]
NO	5.2	TDLAS	2 ppb	Namjou *et al.* [110]
NO	5.2	ICOS	400 ppt (<1 sec)	McCurdy *et al.* [88]
^14^NO and ^15^NO	5.0	CALOS	7 ppt (70 sec)	Heinrich *et al.* [111]
